# The effect of infliximab plus methotrexate on the modulation of inflammatory disease markers in juvenile idiopathic arthritis: analyses from a randomized, placebo-controlled trial

**DOI:** 10.1186/1546-0096-8-24

**Published:** 2010-09-07

**Authors:** Sudha Visvanathan, Carrie Wagner, Joseph C Marini, Daniel J Lovell, Alberto Martini, Ross Petty, Ruben Cuttica, Patricia Woo, Graciela Espada, Marco Gattorno, Maria T Apaz, Eileen Baildam, Anders Fasth, Valeria Gerloni, Pekka Lahdenne, Pierre Quartier, Rotraud Saurenmann, Suzanne Travers, Alan Mendelsohn, Stephen Xu, Edward H Giannini, Nicolino Ruperto

**Affiliations:** 1 Current Address: Hoffmann-La Roche, Nutley, NJ, USA; 2 Centocor Research & Development, Inc., Malvern, PA, USA; 3 Cincinnati Children's Hospital Medical Center, Cincinnati, Ohio, USA; 4 IRCCS G. Gaslini and University of Genoa, Genoa, Italy; 5 British Columbia's Children's Hospital and the University of British Columbia, British Columbia, Canada; 6 Hospital General de Niños Pedro de Elizalde, Buenos Aires, Argentina; 7 Great Ormond St Hospital for Children, London, UK; 8 Hospital de Ninos Ricardo Gutierrez, Buenos Aires, Argentina; 9 Universidad Catolica, Cordoba, Argentina; 10 Royal Liverpool Children's Hospital, Liverpool, UK; 11 University of Gothenberg and The Queen Silvia Children's Hospital, Götenborg, Sweden; 12 Istituto Gaetano Pini, Milano, Italy; 13 Hospital for Children and Adolescents, Helsinki, Finland; 14 Hopital Necker Enfants Malades, Assistance Publique Hopitaux de Paris and Universite Paris-Descartes, Paris, France; 15 University Children's Hospital, Zürich, Switzerland; 16 IRCCS G. Gaslini, Pediatria II, PRINTO, Genoa, Italy

## Abstract

**Background:**

We evaluated the effect of infliximab on markers of inflammation in patients with juvenile idiopathic arthritis (JIA).

**Methods:**

In this randomized, placebo-controlled substudy, 122 patients with JIA received infliximab 3 mg/kg + methotrexate (MTX)(n = 60) or placebo + MTX (n = 62) at weeks 0, 2, and 6. At week 14, patients receiving placebo + MTX crossed over to infliximab 6 mg/kg + MTX; patients receiving infliximab 3 mg/kg + MTX continued treatment through week 44. Sera and plasma from eligible patients receiving infliximab 3 mg/kg + MTX (n = 34) and receiving placebo→infliximab 6 mg/kg +MTX (n = 38) were collected at weeks 0, 2, 14, 16, 28, and 52 and analyzed for inflammatory markers (IL-6, IL-12p40, ICAM-1, MMP-3, VEGF, TNF-α, and CRP).

**Results:**

At week 2, decreases from baseline in IL-6, ICAM-1, MMP-3, TNF-α, and CRP were greater with infliximab versus placebo treatment, and with the exception of CRP, these differences were generally maintained through week 14. The decreases from baseline to week 52 in IL-6, ICAM-1, VEGF, MMP-3, and CRP and increases in IL-12p40 levels were larger in patients receiving placebo→infliximab 6 mg/kg +MTX versus infliximab 3 mg/kg + MTX treatment. Patients receiving infliximab 3 mg/kg+MTX who achieved an American College of Rheumatology Pediatric 30 (ACR-Pedi-30) response had significantly larger decreases from baseline in ICAM-1 (p = 0.0105) and MMP-3 (p = 0.0253) at week 2 and in ICAM-1 (p = 0.0304), MMP-3 (p = 0.0091), and CRP (p = 0.0011) at week 14 versus ACR-Pedi-30 nonresponders.

**Conclusion:**

Infliximab + MTX attenuated several inflammatory markers in patients with JIA; larger decreases in ICAM-1, MMP-3, and CRP levels were observed in ACR-Pedi-30 responders versus nonresponders.

**Trial Registration:**

NCT00036374

## Background

Juvenile idiopathic arthritis (JIA, formerly referred to as polyarticular course juvenile rheumatoid arthritis [JRA]) is the most common chronic rheumatic disorder in children [1,2]. Tumor necrosis factor-alpha (TNF-α) plays an important role in the disease process underlying the chronic inflammation that characterizes JIA. Elevated serum levels of inflammatory markers TNF-α, interleukin (IL)-6, IL-12 and vascular endothelial growth factor (VEGF) have been detected in patients with systemic onset of JIA [3-5]. Increased levels of matrix metalloproteinase (MMP)-1 and MMP-3 [6] and more recently IL-17 [7] have been observed in the synovial fluid of patients with JIA. Further, elevated levels of intercellular cell adhesion molecule (ICAM)-1 and E-selectin in serum have been correlated with active joint count in JIA patients [8]. Thus, different inflammatory processes are perpetuated in the JIA disease process and these may be linked to clinical manifestations.

In the ASPIRE study of adults with early RA, treatment with infliximab plus methotrexate (MTX) resulted in rapid reductions in levels of MMP-3, ICAM-1, IL-8 and TNF-α [9]. While weekly MTX treatment, at parenteral doses up to 15 mg/m^2^, is an effective and safe therapy in JIA [10,11], pediatric patients not responding to MTX may now have other treatment options with the anti-TNF-α therapies etanercept and adalimumab [12,13]. Further, in a recent, small study of JIA patients by Levalampi and colleagues, treatment with infliximab resulted in reductions in levels of C-reactive protein (CRP), adhesion molecules (ICAM-1, E -selectin) and myeloperoxidase [14].

Results from an international, multicenter, randomized, placebo-controlled, double-blind study of the anti-TNF agent infliximab in the treatment of 122 children with persistent polyarticular JRA despite prior MTX, have been reported. While infliximab treatment at doses of 3 mg/kg and 6 mg/kg yielded durable efficacy at 1 year, achievement of the primary clinical endpoint (i.e., American College of Rheumatology Pediatric 30 Definition of Improvement [ACR-Pedi-30 response]) at 3 months did not differ significantly between infliximab 3 mg/kg- and placebo-treated patients [15]. Here we report the results of a subanalysis of this study that was performed to determine the effect of infliximab treatment on select markers of inflammation and assess correlations between changes in the inflammatory markers and changes in ACR-Pedi-30 response status.

## Methods

### Patients and study design

The details of the study design and patient eligibility criteria have been previously described [15]. Briefly, this substudy was conducted as part of a phase 3, double-blind, placebo-controlled study of the safety and efficacy of infliximab in a subset of pediatric patients (age 4-17 years) of adequate weight (≥35 kg) with JIA (defined in the study protocol as JRA). Patients were randomized in a 1:1 ratio to one of two treatment groups. Patients initially receiving placebo + MTX (n = 62) received placebo at weeks 0, 2, and 6, and then crossed over to receive infliximab 6 mg/kg + MTX at weeks 14, 16, 20, and then every 8 weeks through week 44. Patients in the infliximab 3 mg/kg + MTX group (n = 60) received infliximab 3 mg/kg + MTX at weeks 0, 2, 6, 14, 20, and then every 8 weeks through week 44; these patients also received a placebo infusion at week 16 to maintain the study blind. Patients in both groups received concomitant MTX (10 - 15 mg/m^2 ^weekly) throughout the study. Sera and plasma samples were collected at weeks 0, 2, 14, 16, 28, and 52 in patients with an adequate body weight (≥35 kg; 34 patients receiving infliximab 3 mg/kg + MTX and 38 patients receiving placebo/infliximab 6 mg/kg + MTX).

### Biomarker assessments

Plasma levels of IL-6 and serum levels of MMP-3, ICAM-1, IL-12p40, and VEGF were measured using enzyme-linked immunosorbent assay (ELISA) kits (R&D Systems, Minneapolis, MN). TNF-α levels were measured using a Bio-plex™ bead-based sandwich enzyme immunoassay technique (Bio-Rad Laboratories, Hercules, CA). These validated analyses were conducted in a blinded manner at Centocor Ortho Biotech, Inc. Quintiles Laboratories (GA) measured serum CRP concentrations using the Roche Tinaquant assay (Roche Diagnostics, IN).

### Statistical analysis

The median percent changes from baseline in inflammatory marker levels at weeks 2, 14, 16, 28, and 52 were determined. Statistical comparisons were made between patients receiving placebo + MTX who crossed over to receive infliximab 6 mg/kg + MTX and those receiving infliximab 3 mg/kg + MTX using analysis of variance on the van der Waerden scores. Univariate Spearman rank correlations were performed between individual marker levels at baseline.

Correlation analyses were also performed between changes from baseline to week 14 for each biomarker and the number of joints with active arthritis. Baseline levels and changes from baseline to week 2 and week 14 in levels of inflammatory markers were also compared between ACR-Pedi-30 responders and nonresponders at week 14 using analysis of variance on the van der Waerden scores. ACR-Pedi-30 responders were defined as patients achieving an improvement of at least 30% in any 3 of the 6 core variables (i.e., physician global assessment, parent/patient global assessment of overall well-being, functional ability determined by the Childhood Health Assessment Questionnaire [16], number of joints with active arthritis, number of joints with limited range of motion, and erythrocyte sedimentation rate) with no more than one variable worsening by more than 30% [16]. P-values are provided for exploratory purposes and are not adjusted for multiplicity.

## Results

### Baseline characteristics

Baseline patient and disease characteristics were similar between the two randomized treatment groups. In addition, no significant differences were observed between the randomized treatment groups for the median baseline CRP, IL-6, MMP-3, ICAM-1, IL-12p40, VEGF, or TNF-α levels (Table 1). Only 14 patients who received placebo + MTX and 9 patients who received infliximab 3 mg/kg + MTX had TNF-α levels above the lower limit of quantification of the assay, which restricted subsequent data analyses involving this inflammatory marker.

**Table 1:** Disease characteristics and biomarker levels at baseline*

	Placebo→Infliximab 6 mg/kg + MTX	Infliximab 3 mg/kg + MTX
Patients included in substudy	38	34
Age at study entry, yrs	12.7 (3.3)	13.4 (2.8)
Disease characteristics		
Disease duration, yrs	4.03 (3.92)	4.89 (4.4)
Number of joints with active arthritis		
n	38	34
Range	(5.0, 47.9)	(5.0, 44.0)
Median	16.0	13.5
Number of joints with limited range of motion		
n	38	34
Range	(4.0, 48.4)	(2.0, 42.0)
Median	15.0	12.5
Inflammatory markers		
C-reactive protein, mg/dL		
n	38	34
Range	(0.4, 7.4)	(0.4, 10.5)
Median	0.8	0.9
TNF-α (pg/mL)		
n	14	9
Range	(1.6, 30.9)	(1.6, 24.4)
Median	1.6	1.6
IL-6 (pg/mL)		
n	28	27
Range	(0.4, 93.7)	(0.1, 79.3)
Median	8.2	3.4
MMP-3 (ng/mL)		
n	32	32
Range	(2.7, 390.2)	(0.8, 570.0)
Median	39.3	40.3
ICAM-1 (ng/mL)		
n	34	32
Range	(200.2, 767.9)	(18.6, 1059.2)
Median	394.0	375.4
VEGF (ng/mL)		
n	33	32
Range	(83.3, 1535.2)	(29.8, 1447.0)
Median	440.2	317.8
IL-12p40 (pg/mL)		
n	34	30
Range	(15.5, 357.9)	(15.6, 357.9)
Median	84.3	95.6

Values are presented as mean (SD) unless otherwise noted.

* Only patients with biomarker values above the lower limit of quantification of specific assays were included in analyses, which accounts for differences between numbers of patients sampled for each inflammatory marker.

MTX, methotrexate; IL-6, interleukin-6; MMP-3, matrix metalloproteinase-3; ICAM-1, intracellular cell adhesion molecule-1; VEGF, vascular endothelial growth factor; IL-12p40, interleukin 12; TNF-α, tumor necrosis factor-α.

Correlations between inflammatory markers at baseline indicated that the strongest correlations were between CRP and IL-6 (r = 0.72, p < 0.0001), MMP-3 and CRP (r = 0.53, p < 0.0001), and MMP-3 and IL-6 (r = 0.52, p = 0.0001). Weaker correlations were also observed between MMP-3 and VEGF, TNF-α and ICAM-1, TNF-α and IL-12p40, and CRP and ICAM-1 (data not shown). Further, baseline IL-6 and ICAM-1 levels significantly correlated with the number of joints with active arthritis at baseline (r = 0.37, p = 0.0059; r = 0.32, p = 0.0096; respectively).

### Changes in inflammatory marker levels during the placebo-controlled period assessed at weeks 2 and 14

**Figure 1:**
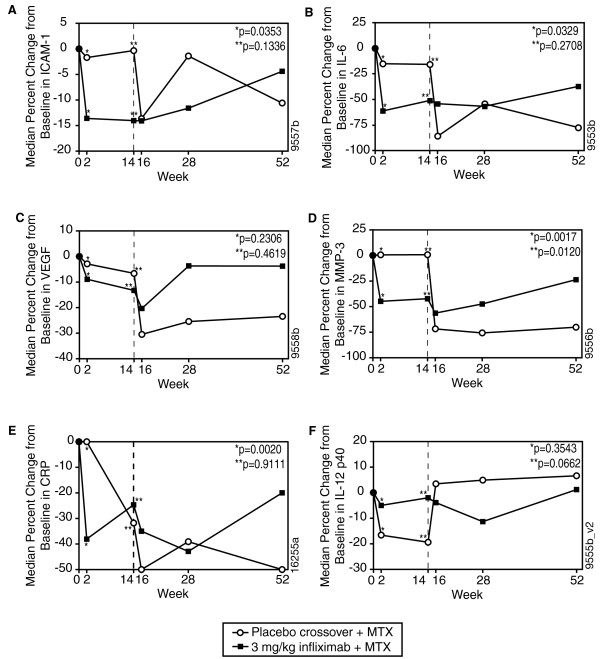
**Change in median levels of serum inflammatory markers over time in patients who received infliximab 3 mg/kg + MTX or placebo + MTX through week 14 with a crossover to infliximab 6 mg/kg + MTX for ICAM-1 (A), IL-6 (B), VEGF (C), MMP-3 (D), CRP (E), and IL-12p40 (F)**. MTX, methotrexate; ICAM-1, intracellular cell adhesion molecule-1; IL-6, interleukin-6; VEGF, vascular endothelial growth factor; MMP-3, matrix metalloproteinase-3; CRP, C-reactive protein; IL-12p40, interleukin 12.

At week 2, the median percent decreases from baseline in ICAM-1 (-13.6% vs. -1.7%; p = 0.0353), IL-6 (-61.6% vs. -15.4%; p = 0.0329), and CRP (-38.1% vs. 0.0%; p = 0.0020) were greater in patients receiving infliximab 3 mg/kg + MTX than in those receiving placebo + MTX. A 44.9% decrease from baseline to week 2 in MMP-3 was also observed in patients receiving infliximab 3 mg/kg + MTX, compared with an increase of 0.6% in patients receiving placebo + MTX (p = 0.0017) (Figures 1A, 1B, 1D, and 1E). In contrast, a larger median percent decrease in IL-12p40 levels was observed in the placebo + MTX group (-16.6%) than in the infliximab 3 mg/kg + MTX group (-5.0%; p = NS).

Median reductions were numerically higher at week 14 in patients receiving infliximab 3 mg/kg + MTX compared with those receiving placebo + MTX for ICAM-1 (-14.4 vs. -0.4; p = NS), IL-6 (-51.6 vs. -16.2; p = NS), VEGF(-13.3 vs. -6.7; p = NS), and MMP-3(-42.4 vs. 0.7; p = 0.0120) levels (Figures 1A-D. The decreases in CRP and IL-12p40 levels observed at week 14 were greater in patients receiving placebo + MTX (-31.8 and -19.4, respectively) than in those receiving infliximab 3 mg/kg + MTX (-24.7 and -2.0, respectively), but these changes were not significant (p = 0.9111 and 0.0622, respectively; Figures 1E, 1F).

### Change in inflammatory marker levels during the crossover period assessed at weeks 16, 28, and 52

By week 20, at which point all patients were receiving either 3 or 6 mg/kg infliximab + MTX, reduced levels of inflammatory markers were observed in both treatment groups for ICAM-1, IL-6, VEGF, MMP-3 and CRP (Figures 1A-E. Levels of IL-12p40 had also declined in the infliximab 3 mg/kg + MTX group, but showed an increase (3.5%) at week 16 in patients receiving placebo who crossed over to receive infliximab 6 mg/kg + MTX (Figure 1F).

Variable changes from baseline were observed across the inflammatory markers in patients receiving infliximab 3 mg/kg + MTX or infliximab 6 mg/kg + MTX at weeks 28 and 52. While inflammatory marker levels tended to rebound somewhat over time in the infliximab 3 mg/kg group, ICAM, IL-6, VEGF, MMP-3 and CRP levels at week 52 were generally below levels observed at baseline (Figures 1A-E). Conversely, IL-12p40 levels were slightly increased from baseline to week 52 (Figure 1F). The decreases from baseline to week 52 in IL-6, ICAM-1, MMP-3, and CRP and increases in IL-12p40 levels were larger in patients receiving placebo + MTX who crossed over to receive infliximab 6 mg/kg + MTX as compared with patients receiving infliximab 3 mg/kg + MTX. Only the differences in IL-6 (-77.8% vs. -37.7%; p = 0.0098, respectively) and MMP-3 (-70.2% vs. -23.8%; p = 0.0258, respectively) were statistically significant (data not shown).

### Associations between inflammatory marker levels and clinical response as assessed by ACR-Pedi-30 response and the number of joints with active arthritis

At week 2, among patients receiving placebo + MTX, significantly larger median percent decreases in IL-6 (-49.6% vs. 6.8%; p = 0.0344) were observed in patients who were ACR-Pedi-30 responders as compared with ACR-Pedi-30 nonresponders. These results indicated a significant association between changes in IL-6 levels and ACR-Pedi-30 response in patients treated with placebo + MTX (Table 2). Significantly larger median percent decreases in ICAM-1 (p = 0.0105) and MMP-3 (p = 0.0253) were also observed among ACR-Pedi-30 responders when compared with nonresponders in the infliximab 3 mg/kg + MTX group (Table 2).

**Table 2:** Median percent change in biomarker levels from baseline by ACR-Pedi-30 responder status

	Placebo +MTX					Infliximab 3 mg/kg +MTX				
		
	ACR-Pedi-30 Responder		ACR-Pedi-30 Nonresponder		P-value	ACR-Pedi-30 Responder		ACR-Pedi-30 Nonresponder		P-value
Biomarker	N	Median	N	Median		N	Median	N	Median	
***Week 2***										
CRP	29	-11.1	28	0.0	NS	39	-54.3	19	0.0	NS
ICAM-1	13	-10.6	18	4.2	NS	21	-24.5	9	-3.8	0.0105
IL-12p40	16	-16.6	16	-16.2	NS	20	-9.8	9	-5.0	NS
IL-6	12	-49.6	12	6.8	0.0334	17	-57.1	9	-66.1	NS
MMP-3	11	-0.6	16	-0.8	NS	21	-60.3	9	-4.8	0.0253
TNF-α	16	0.0	18	0.0	NS	24	0.0	9	0.0	NS
VEGF	12	4.6	18	-5.6	NS	22	-10.6	8	-2.5	NS
***Week 14***										
CRP	29	-40.0	27	-6.3	NS	39	-55.0	19	0.0	0.0011
ICAM-1	13	-8.0	18	12.4	NS	22	-21.7	8	4.8	0.0304
IL-12p40	15	-19.4	17	-18.5	NS	21	-4.0	9	10.8	NS
IL-6	11	-16.2	14	-20.6	NS	18	-62.6	8	-26.5	NS
MMP-3	11	21.7	17	-21.7	NS	22	-55.5	8	8.2	0.0091
TNF-α	16	-4.8	18	0.0	NS	24	0.0	9	0.0	NS
VEGF	11	13.2	18	-6.7	NS	23	-18.9	7	18.7	NS

ACR-Pedi-30, American College of Rheumatology Pediatric 30 Definition of Improvement; MTX, methotrexate; NS, non-significant; CRP, C-reactive protein; IL-6, interleukin-6; MMP-3, matrix metalloproteinase-3; ICAM-1, intracellular cell adhesion molecule-1; VEGF, vascular endothelial growth factor; IL-12p40, interleukin 12; TNF-α, tumor necrosis factor-α.

At week 14, in patients receiving infliximab 3 mg/kg + MTX, ACR-Pedi-30 responders had larger median percent reductions than ACR-Pedi-30 nonresponders in ICAM-1 (-21.7% vs. 4.8%; p = 0.0304), MMP-3 (-55.5% vs. 8.2%; p = 0.0091), and CRP (-55.0% vs. 0.0%; p = 0.0011; Table 2).

Also, at week 14, decreases in IL-6, VEGF, and CRP levels correlated with the number of joints with active arthritis in patients treated with infliximab 3 mg/kg + MTX (r = 0.52, p = 0.005; r = 0.44, p = 0.011; r = 0.29, p = 0.027; respectively). In the placebo + MTX group, changes in ICAM-1 and CRP levels were significantly correlated with the number of joints with active arthritis at week 14 (r = 0.64, p < 0.0001; r = 0.38, p = 0.004; respectively) (data not shown).

## Discussion

JIA involves an inflammatory process that if unabated will eventually lead to bone and cartilage damage. Thus we examined markers associated with inflammation (IL-6, ICAM-1, MMP-3, VEGF, IL-12p40, TNF-α, and CRP) that may be modulated by infliximab + MTX therapy. Although the primary clinical endpoint for this study was not achieved, results of the current analysis indicate that significant reductions from baseline in IL-6, ICAM-1, MMP-3, and CRP levels were observed as early as week 2 and sustained through week 14 of treatment with infliximab 3 mg/kg + MTX, relative to treatment with placebo + MTX. Similar findings were reported by Levalampi and colleagues, who reported reductions in CRP, IL-6 and ICAM-1 levels by week 6 in JIA patients treated with infliximab [14]. Taken together, these findings suggest that early decreases in these biomarkers are an indication of disease modulation.

Further, in the current study, larger decreases in IL-6, ICAM-1, MMP-3 and CRP and increases in IL-12p40 levels were observed in patients receiving placebo + MTX who crossed over to receive infliximab 6 mg/kg + MTX relative to treatment with infliximab 3 mg/kg + MTX. Increased IL-12 and interferon gamma (IFNγ) mRNA expression has previously been detected in synovial tissue from JIA patients, which suggests that IL-12 may have an important role in this disease [17]. The increase in IL-12 levels in infliximab-treated patients may reflect a shift in the disease processes resulting from substantial reduction of peripheral TNF-α levels. In patients treated with infliximab 3 mg/kg + MTX, those who achieved an ACR-Pedi-30 response showed significant reductions in ICAM-1, MMP-3, and CRP levels compared with those who did not achieve an ACR-Pedi-30 response. Further, decreases in IL-6, VEGF, and CRP levels were correlated with the number of joints with active arthritis. These results suggest that ICAM-1, MMP-3, IL-6, VEGF, and CRP might be useful surrogate markers to monitor improvement in the signs and symptoms of JIA and joints with active arthritis after initiation of treatment with infliximab + MTX therapy.

This biomarker substudy has several limitations. First, the substudy was designed as an exploratory assessment of changes in inflammatory biomarker levels after infliximab 3 mg/kg + MTX therapy versus treatment with placebo + MTX therapy in patients with JIA. Second, the patients evaluated in both treatment groups were limited to those who were of adequate body weight (≥35 kg) and analyses were based only on patients with levels of inflammatory markers above the lower limit of quantification for each assay. Further, median TNF-α levels were not substantially elevated, which indicates that systemic levels of this biomarker are low in this evaluated patient population. An additional limitation of this substudy was the inability to measure free TNF-α after infliximab treatment since the available commercial TNF-α assays were not designed to distinguish "free" from "infliximab-bound" TNF-α. These study limitations may have limited our ability to detect potentially significant associations between biomarker changes and improvement in clinical signs and symptoms in either the placebo + MTX crossover or infliximab 3 mg/kg + MTX groups, or to determine if these trends would be the same in patients who weighed less than 35 kg.

## Conclusions

Findings of this biomarker substudy suggest that changes in ICAM-1, MMP-3, IL-6, VEGF and CRP levels may be useful markers to evaluate response to infliximab therapy in patients with JIA and thus may enable early identification of patients who could benefit from this treatment. Larger studies will be required to confirm the current preliminary findings and establish the relevance of these markers in predicting changes in signs and symptoms, as well as structural damage, of JIA.

## Competing interests

SV, CW, JCM, ST, AlaM and SX were all employees of Johnson and Johnson at the time of the study and owners of Johnson and Johnson stock. NR, DJL, PW, RP, AlbM and EHG have received honoraria and/or consulting fees from Centocor for serving as members of the study steering committee. However they did not receive any funding related to the current paper. DJL also served as a consultant for Centocor, Abbott, Amgen, Bristol Meyers Squibb, Hoffman La-Roche, Novartis, Pfizer, Regeneron, Roche, and XOMA. He is on the Speakers Bureau for Wyeth, Amgen DSMB chairman, and on the Editorial board for Arthritis Care and Research. He also received grant/research support from NIH and FDA. RS served as an advisory board member for Remicade Switzerland and received payments of less than $2,000/year for participation in advisory board meetings. PL received consultancies, congress grants, and speaking fees, all less than $10,000 from Aventis, Schering-Plough, Abbott, and Wyeth and received compensation for her role as a PI in a Centocor-sponsored study of JIA-infliximab in Helsinki, Finland.

All other authors declare that they have no competing interests.

## Authors' contributions

SV participated in acquisition of the data, analysis and interpretation of the data, and manuscript preparation. CW and JCM participated in analysis and interpretation of the data and manuscript preparation. DJL and NR participated in study design, acquisition of the data, analysis and interpretation of the data, manuscript preparation, and study logistics and were part of the steering committee. AlbM and EHG participated in study design, analysis and interpretation of data, and manuscript preparation. RP, RC, PW, GE, MG, MA, EB, AF, VG, PL, PQ, and RS participated in acquisition of the data and preparation of the manuscript. AlaM participated in acquisition of the data, analysis and interpretation of the data, and manuscript preparation. ST participated in study design, acquisition of the data, analysis and interpretation of the data, and manuscript preparation. SX participated in analysis and interpretation of the data and helped to draft the manuscript. All authors read and approved the final manuscript.
